# Methodological issues in assessing changes in costs pre- and post-medication switch: a schizophrenia study example

**DOI:** 10.1186/1478-7547-7-11

**Published:** 2009-05-27

**Authors:** Douglas E Faries, Allen W Nyhuis, Haya Ascher-Svanum

**Affiliations:** 1 Lilly USA, LLC, Indianapolis, Indiana, USA; 2 Eli Lilly and Company, Indianapolis, Indiana, USA

## Abstract

**Background:**

Schizophrenia is a severe, chronic, and costly illness that adversely impacts patients' lives and health care payer budgets. Cost comparisons of treatment regimens are, therefore, important to health care payers and researchers. Pre-Post analyses ("mirror-image"), where outcomes prior to a medication switch are compared to outcomes post-switch, are commonly used in such research. However, medication changes often occur during a costly crisis event. Patients may relapse, be hospitalized, have a medication change, and then spend a period of time with intense use of costly resources (post-medication switch). While many advantages and disadvantages of Pre-Post methodology have been discussed, issues regarding the attributability of costs incurred around the time of medication switching have not been fully investigated.

**Methods:**

Medical resource use data, including medications and acute-care services (hospitalizations, partial hospitalizations, emergency department) were collected for patients with schizophrenia who switched antipsychotics (n = 105) during a 1-year randomized, naturalistic, antipsychotic cost-effectiveness schizophrenia trial. Within-patient changes in total costs per day were computed during the pre- and post-medication change periods. In addition to the standard Pre-Post analysis comparing costs pre- and post-medication change, we investigated the sensitivity of results to varying assumptions regarding the attributability of acute care service costs occurring just after a medication switch that were likely due to initial medication failure.

**Results:**

Fifty-six percent of all costs incurred during the first week on the newly initiated antipsychotic were likely due to treatment failure with the previous antipsychotic. Standard analyses suggested an average increase in cost-per-day for each patient of $2.40 after switching medications. However, sensitivity analyses removing costs incurred post-switch that were potentially due to the failure of the initial medication suggested decreases in costs in the range of $4.77 to $9.69 per day post-switch.

**Conclusion:**

Pre-Post cost analyses are sensitive to the approach used to handle acute-service costs occurring just after a medication change. Given the importance of quality economic research on the cost of switching treatments, thorough sensitivity analyses should be performed to identify the impact of crisis events around the time of medication change.

## Background

Pre-Post (or "mirror-image") designs are studies where one compares outcomes prior to and after some event – such as a medication change. The focus here is on pre-post comparisons of economic outcomes from patients switching medication treatment for schizophrenia. In long term treatment for schizophrenia, treatment switching is common, and there is a great need for information on treatment options for patients requiring a medication switch [1-3]. Due to the large impact of schizophrenia care on health care payer budgets, the comparison of treatments on economic outcomes has been the focus of much research [4,5]. Understanding if the costs of care decrease upon a change to a particular medication is often a research question of interest that is assessed using pre-post analyses. Hudson and colleagues [6] reviewed 10 economic outcome studies of antipsychotic medications using various pre-post designs and reported mixed results. More recently, pre-post assessments of costs were reported for patients with schizophrenia switching to amisulpride [7], switching between risperidone and olanzapine [8,9] and switching from typicals to atypicals [10].

Health-care decision makers often utilize observational data to assess economic outcomes [11,12]. In comparison to clinical trials, observational research provides broader generalizability due to its utilization of cost and medical resource data which is both real-world and less influenced by protocol-required visits, compliance, and other procedures [11,13]. However, observational data is subject to selection bias that complicates comparisons between nonrandomized groups of patients [14]. One advantage of Pre-Post analyses is that they allow for 'within-patient analyses' (each patient is their own control) – thus avoiding the need to adjust for selection bias that complicates comparisons between (nonrandomized) groups of patients in naturalistic research.

Pre-post analyses have several important disadvantages that have been well described in the literature [15]. These include regression to the mean, time period bias, lack of a control group, and asymmetrical treatment durations. In usual clinical practice settings for the treatment of schizophrenia and other episodic disease states, treatment changes are typically made during a time of symptom exacerbation or a relapse which may require acute care services. Thus, on average, one might expect symptoms to be ameliorated and costs to decrease from that time point. Pre-post analyses compare outcomes that occurred at different times on the same patient under different treatment conditions. Inferring that differential outcomes are due to treatment differences requires one, among other things, to assume no period/time effect, which is especially problematic in the study of episodic disease states such as schizophrenia.

Pre-post analyses are not, in general, suited to address broader questions of interest requiring multiple comparison groups. For instance, in the treatment of schizophrenia one might be interested in evaluating the multiple treatment options at the point of medication change, including switching to one of several medications or changing the dose of the current medication. One option in such instances is to utilize randomization to several treatment options for comparison at the time point where patients were in need of a medication change – as was done in the Clinical Antipsychotic Trials of Intervention Effectiveness (CATIE) [1,16,17]. In naturalistic settings, the duration of each (pre- and post-) treatment varies – thus raising challenges when comparing outcomes based on differing treatment durations. While one can just assess a set period of time, pre- and post-switch, the effect of a drug may differ in the first few weeks of treatment relative to a later period of treatment.

Another limitation of pre-post designs for cost comparisons that has received less attention is the issue of costs incurred while on treatment B (the medication switched to) which may have been due to the failure of treatment A (the initial medication). For instance, while there are many reasons for switching treatment, the most common is lack of efficacy [18], which includes patients who relapse – leading to crisis management such as a hospitalization where treatment A is discontinued and treatment B is first initiated in the hospital. Costs for the management of this relapse may continue for some time after discontinuation of treatment A. While this issue has been raised [15], this is especially important for economic analyses because hospitalizations and the cost of relapse represent the largest portion of health care costs for patients with schizophrenia [19-22].

Our objective in this paper is to highlight the issue of overlapping or carry-over costs from a failed prior treatment onto the next treatment in pre-post medication switch analyses. We accomplish this by examining the sensitivity of pre-post analyses of changes in costs of care to various assumptions regarding the attributability of acute-care service costs occurring immediately after a medication switch. Data from a naturalistic schizophrenia study is utilized for the analysis.

## Methods

### Study Design

Data for this post-hoc analysis came from a cost-effectiveness study of first-line treatment with atypical and conventional antipsychotics over a 1-year period [23,24]. This randomized, naturalistic, open-label trial was conducted from May 1998 through September 2001 at 21 sites in 15 states in the United States. This study used broad eligibility criteria. Patients aged 18 years or older with a DSM-IV diagnosis of schizophrenia, schizoaffective, or schizophreniform disorder were included, provided they scored ≥ 18 on the Brief Psychiatric Rating Scale (BPRS) [25]. No patient was excluded because of comorbid substance abuse disorder or other psychiatric or medical comorbidities. Patients were randomly allocated to a first-line treatment group: 1 of 2 atypical antipsychotics (n = 450) or a conventional first-generation antipsychotic of each physician's choosing (n = 214). Barring clinically significant adverse events, patients remained on their initial medications for at least 8 weeks, after which they could change medications if a switch was clinically warranted. Medications could also be discontinued, or their doses altered, at each physician's discretion.

### Definitions and Measures

Patients were defined as having switched medications if they discontinued the medication to which they were initially randomized and then initiated therapy with a different antipsychotic within 14 days. Acute-care services were defined as hospitalizations, partial hospitalizations, and visits to emergency departments (EDs). Data on each patient's use of such services were systematically abstracted from medical records, patient self-reports, and the study sites' administrative databases. Cost estimates applied to each unit of resource use have been previously described in detail [24]. In brief, Medicare public data were used as cost benchmarks for units of specific services and applied to the collected resource use data to obtain costs of care for each patient. Medication costs were based on 2001 average wholesale prices, discounted 15% to reflect real-world costs [24].

### Analysis Methods

To examine the impact of carry-over (overlapping) costs, we analyzed switching data using 4 different approaches – each making different assumptions regarding the attributability of costs and timing of incurred costs potentially due to the initial (pre) treatment. To standardize the 2 evaluation periods to a common duration, we chose to evaluate the last 60 days on the Pre-treatment and the 1st 60 days on the Post-treatment. The goal was to use a short enough duration to avoid deletion of patients from the analysis (or imputation of missing data) who only had short durations in the study pre- and/or post-switch and long enough to provide a more stable assessment of costs while on the medication. As secondary analyses, we utilized a 30-day window. Costs in the post-period were computed regardless of additional subsequent switches in medications. Costs are presented on an average daily basis to allow for comparisons across methods on a standard time scale. The 4 analysis approaches were:

#### Standard Pre-Post

Costs incurred during the 60 days leading up to the last day on the initial medication are defined as 'Pre' period costs. Costs incurred starting from the day after the initial medication was discontinued for the next 60 days are defined as 'Post' period costs.

#### Remove Overlapping Acute Crisis Costs from Post Period

Costs of acute care services that began while on the initial medication, but continued into the 'Post' medication period, were excluded from the analysis. Otherwise, calculations were the same as for the Standard Pre-Post Switch Analysis.

#### Exclude first 2 weeks of the Post Period

Calculations follow the Standard Pre-Post Switch analysis except that the first 14 days of the Post period were excluded from the calculations. The 2-week cutoff was selected as most hospitalization durations were less than 14 days, so the impact of hospitalizations beginning in the 'pre' period would be addressed and since research suggests that impact of new medication could be observable by 2 weeks [26-28].

#### Assign first 2 weeks of Post Costs to Pre Medication

Calculations follow the Standard Pre-Post Switch analysis except that costs occurring in the first 14 days in the post period are assigned to the Pre-treatment period.

Paired differences in costs (Pre-Post) were compared statistically (with a null hypothesis of no change in mean costs) using both a paired t-test and, due to the skewness of the cost data, a nonparametric bootstrapping approach. The bootstrapping approach was utilized as we desired a nonparametric approach due to the anticipated skewed cost data and a test for differences in means as opposed to medians. Means are the appropriate parameter of interest as health care payers are responsible for the cost of the entire population, and N-times-the-median may not be representative of the total costs given skewed distributions [29,30].

## Results

A total of 664 patients were randomized, though 13 never started on their assigned medication [24]. Of the 651 analyzable patients, 191 (29.3%) switched antipsychotics at some point during the 1-year study; 144 patients switched antipsychotics AND had information in the study from 30 days pre- to 30 days post-switch available for analysis; and 105 patients switched antipsychotics and had information from 60 days pre- and post-switch available for analysis. Thus, analyses here are based on 105 patients. Analysis with the 30-day requirement (n = 144) provided similar results. Of the 460 patients who did not switch antipsychotics during the study, 31.7% were early discontinuers and did not complete 1 year on study. It is anticipated that some of these patients may have changed medications outside of the clinical study. Thus, actual rates of switching is anticipated to be greater than 29.3%, but data on these potential switching patients was not available.

Table 1 summarizes the patient characteristics at study enrollment. Patient characteristics of the switchers were relatively similar to the nonswitchers except for having a greater proportion of females, lower rates of substance abuse, and prior-year hospitalizations.

**Table 1:** Patient Characteristics at Study Enrollment

	Analyzed Switchers ^a^	All Switchers	Non-Switchers
N	105	191	460

Male, %	49.5 ^b^	54.5 ^c^	66.7 ^b, c^

Age, mean (SD)	43.8 (13.2)	42.8 (12.5)	42.9 (11.9)

Age of onset, mean (SD)	22.5 (8.5)	22.1 (8.8)	22.3 (9.8)

Unemployed, (%)	82.9	81.2	81.1

Race, (%)			

Caucasian	58.1	56.0	53.7

African descent	34.3	33.0	33.9

Other	7.6	11.0	12.4

Diagnosis, (%)			

Schizophrenia	65.7	63.4	66.1

Schizoaffective	27.6	28.3	25.9

Schizophreniform	6.7	8.4	8.0

Comorbidities (%)			

MDD	14.3	14.7	13.9

Anxiety	3.8	2.6	6.3

Substance Abuse	20.2^b^	27.4	33.7 ^b^

Baseline PANSS total, mean (SD)	84.6 (19.3)	84.5 (18.8)	87.8 (20.3)

Hospitalization in year prior to enrollment	22.1^b^	27.1	32.3^b^

Abbreviations: PANSS, positive and negative syndrome scale; MDD, Major Depressive Disorder

^a^Switchers with 60 days pre-switch and 60 days post-switch information available

^b^p-value < .05 for comparison between analyzed switchers and nonswitchers

^c^p-value < .05 for comparison between all switchers and nonswitchers

**Figure 1:**
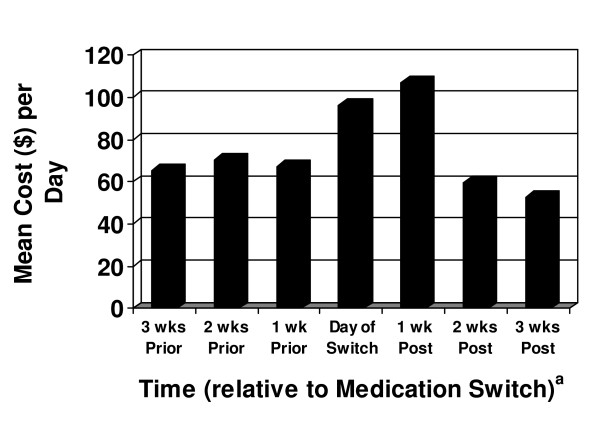
**Mean Total Cost Per Day Relative to the Time of Switching Antipsychotics (N = 105)**. ^a^For example, the '3 wks Prior' column indicates the average daily total cost of care for patients during the 15 to 21 days (inclusive) prior to the medication switch.

Figure 1 summarizes the average total cost per patient per day relative to the time of the medication switch. Costs clearly increased on the day of switch and remained high through the first week after switch. Costs from acute crisis treatment such as hospitalizations, partial hospitalizations, and emergency room visits that started while on the initial medication and continued after the medication switch made up 58 percent of the costs during the first week post-switch and 26% of the costs in the second week post-switch. While one cannot be completely sure of attributability in such cases, given the timing of the hospital admissions (or initiation of partial hospitalization treatment) such costs were determined to be more appropriately linked to failure of the first treatment rather than the lack of effectiveness of the next treatment.

**Figure 2:**
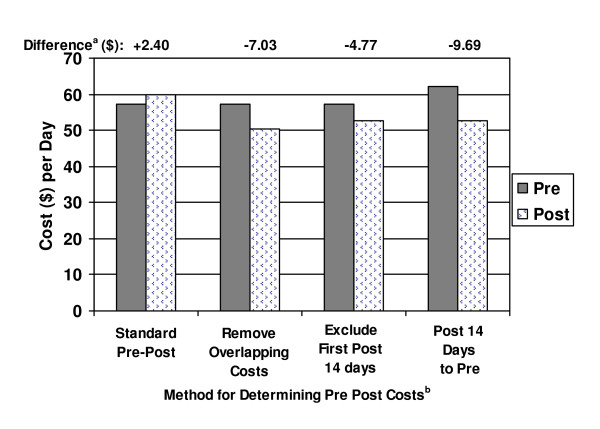
**Pre-Post Switch Cost Analyses (N = 105)**. ^a^None of these differences were statistically significant. ^b^Definitions: Standard Pre-Post: Costs incurred during the 60 days leading up to the last day on the initial medication are defined as 'Pre' period costs. Costs incurred starting from the day after the initial medication was discontinued for the next 60 days are defined as 'Post' period costs. Removed Overlapping Costs (Remove Overlapping Acute Crisis Costs from Post Period): Costs of acute care services that began while on the initial medication but continued into the 'Post' medication period were excluded from the analysis. Otherwise, calculations were the same as for the Standard Pre-Post analysis. Excluded first 2 weeks Post (Exclude first 2 weeks of the Post Period): Calculations follow the Standard Pre-Post Switch analysis except that the first 14 days of the 'Post' period were excluded from the calculations. Post 14 Days to Pre (Assign first 2 weeks of Post Costs to Pre Medication): Calculations follow the Standard Pre-Post Switch analysis except that costs occurring in the first 14 days in the post period are assigned to the Pre-treatment period.

Figure 2 summarizes the 60-day pre and post costs using each of the 4 methods. Standard Pre-Post analyses suggest an average increase in cost per day for each patient of $2.40 after switching medications. However, the other analyses suggested a decrease in the range of $4.77 – $9.69 per day post-switch. The $4.77 decrease was estimated when the first 14 days in the post period was excluded and the $9.69 decrease was estimated when the entire first 14 days in the post period was considered as part of the pre period. The difference in the estimated change in daily costs, pre to post, varied by over $12 per day.

None of the changes in costs were statistically significant from zero, and confidence intervals on the differences in pre-post cost changes for the 4 approaches are large and overlapping. For example, the 95% two-sided confidence interval for the standard approach was (-$12.12, $18.69) and for assigning the first 14 days of post costs to the pre period was (-$24.55, $5.71). Due to the high variability in total costs of care, such studies must be fairly large in order to have enough power for relevant cost comparisons. However, the focus here was not on establishing whether a change in costs occurred, but to compare the methodologies.

## Discussion

The current analyses have demonstrated that assumptions about costs potentially attributable to failure of the first medication can have a directional impact on results when assessing the economic impact of changing medications using a pre-post analysis. The difference in estimated changes in daily costs in this study varied by over $12 per day across the methods utilized (between -$9.69 and +$2.40). Acute care service costs, which is the largest cost component in the treatment of schizophrenia, is often associated with relapse and with changes in medications, and thus must be considered carefully. Given the variation in results depending upon the attribution of crisis care costs around the time of switch, sensitivity analyses are critical to proper understanding of such data.

Caution regarding the analysis of costs occurring around the time of switch should not be limited to pre-post designs. For instance, in clinical trials patients are typically discontinued at the point at which they have a medication change. This potentially ignores a substantial amount of the treatment costs which would then occur outside of the purview of the trial. In practical clinical trials, where patients are allowed to change medications and remain in the study, it is common to conduct an analysis including only the period of time in which the patient was on the medication of interest [31]. Once again, this analysis could be misleading due to missing the potentially large costs occurring just after discontinuing the medication that should be owned by the initial medication.

The availability of data from a control group may address some of the issues associated with pre-post analyses. For instance, pre- and post-switch data for patients treated with a comparison medication would allow for additional methodological approaches to be used. Fu and colleagues [32] compare the use of a difference-in-differences approach along with propensity score-based methodology in such settings. One may also benefit by considering a pre-post analysis in comparison to assessing treatment effects in a crossover design. Without a control group, one simply has a single arm of a standard 2 × 2 crossover trial. The availability of a comparator group may also allow the use of crossover related methods [33].

This study has several limitations. First, the number of patients switching was relatively small for comparisons of costs. Replication with a larger sample is needed due to the high variability in cost data. Next, we were unable to fully collect the reason for switching, which may indeed be associated with costs incurred around the time of switch. Lastly, about one-third of the nonswitchers discontinued early from the study. Some proportion of these patients likely switched medications outside of the purview of the trial, but we were unable to collect post-switch economic data for these patients. At baseline, these early discontinuers were more likely to be male; had more severe symptoms (as measured by the BPRS); and had a higher rate of prior hospitalization, substance abuse, and comorbid anxiety as compared to the switchers in this study. Thus, it is not clear if the results observed here are generalizable to such patients.

## Conclusion

Pre-post cost analyses ("mirror-image") are complicated by costs incurred post-switch that are likely due to the failure of the initial treatment. Such costs are not ignorable and can have substantial impact on the analyses. Sensitivity analyses should be performed to identify the impact of such costs.

## Competing interests

This work was sponsored by Eli Lilly and Company. The authors are full-time employees and minor shareholders of Eli Lilly and Company.

## Authors' contributions

DEF participated in the conception and design of the study, acquisition of data, analysis and interpretation of data, and drafting of the manuscript. AWN participated in the acquisition of data, interpretation of data, and critical revisions of the manuscript. HA-S participated in the conception and design of the study, interpretation of data, and drafting of the manuscript. All authors gave approval of the final version of the manuscript.

## References

[B1] Lieberman JA, Stroup TS, McEvoy JP, Swartz MS, Rosenheck RA, Perkins DO, Keefe RS, Davis SM, Davis CE, Lebowitz BD, Severe J, Hsiao JK, Clinical Antipsychotic Trials of Intervention Effectiveness (CATIE) Investigators (2005). Effectiveness of antipsychotic drugs in patients with chronic schizophrenia. N Engl J Med.

[B2] Essock SM, Covell NH, Davis SM, Stroup TS, Rosenheck RA, Leiberman JA (2006). Effectiveness of switching antipsychotic medications. Am J Psychiatry.

[B3] Ascher-Svanum H, Zhu B, Faries D, Landbloom R, Swartz M, Swanson J (2006). Time to discontinuation of atypical versus typical antispsychotics in the naturalistic treatment of schizophrenia. BMC Psychiatry.

[B4] Duggan M (2005). Do new prescription drugs pay for themselves? The case of second-generation antipsychotics. J Health Econ.

[B5] Polsky D, Doshi JA, Bauer MS, Glick HA (2006). Clinical trial-based cost-effectiveness analyses of antipsychotic use. Am J Psychiatry.

[B6] Hudson TJ, Sullivan G, Feng W, Owen RR, Thrush CR (2003). Economic evaluations of novel antipsychotic medications: a literature review. Schizophr Res.

[B7] Surguladze S, Patel A, Kerwin RW, Knapp M, Travis MJ (2005). Cost analysis of treating schizophrenia with amisulpride: naturalistic mirror image study. Prog Neuropsychopharmacol Biol Psychiatry.

[B8] Zhao Z, Namjoshi M, Barber BL, Loosbrock DL, Tunis SL, Zhu B, Breier A (2004). Economic outcomes associated with switching individuals with schizophrenia between risperidone and olanzapine: findings from a large US claims database. CNS Drugs.

[B9] Takahashi H, Kamata M, Yoshida K, Ishigooka J, Higuchi H (2006). Switching to olanzapine after unsuccessful treatment with risperidone during the first episode of schizophrenia: an open-label trial. J Clin Psychiatry.

[B10] Park S, Ross-Degnan D, Adams AS, Sabin J, Kanavos P, Soumerai SB (2005). Effect of switching antipsychotics on antiparkinsonian medication use in schizophrenia: population-based study. Br J Psychiatry.

[B11] Haro JM, Kontodimas S, Negrin MA, Ratcliffe M, Suarez D, Windmeijer F (2006). Methodological aspects in the assessment of treatment effects in observational health outcomes studies. Appl Health Econ Health Policy.

[B12] Garrison LP, Neumann PJ, Erickson P, Marshall D, Mullins CD (2007). Using real-world data for coverage and payment decisions: the ISPOR Real-World Data Task Force report. Value Health.

[B13] Roy-Byrne PP, Sherbourne CD, Craske MG, Stein MB, Katon W, Sullivan G, Means-Christensen A, Bystritsky A (2003). Moving treatment research from clinical trials to the real world. Psychiatr Serv.

[B14] D'Agostino RB, D'Agostino RB (2007). Estimating treatment effects using observational data. JAMA.

[B15] Gianfrancesco F, Wang RH, Mahmoud R, White R (2002). Methods for claims-based pharmacoeconomic studies in psychosis. Pharmacoeconomics.

[B16] Stroup TS, Lieberman JA, McEvoy JP, Swartz MS, Davis SM, Capuano GA, Rosenheck RA, Keefe RS, Miller AL, Belz I, Hsiao JK, CATIE Investigators (2007). Effectiveness of olanzapine, quetiapine, and risperidone in patients with chronic schizophrenia after discontinuing perphenazine: a CATIE study. Am J Psychiatry.

[B17] McEvoy JP, Lieberman JA, Stroup TS, Davis SM, Meltzer HY, Rosenheck RA, Swartz MS, Perkins DO, Keefe RS, Davis CE, Severe J, Hsiao JK, CATIE Investigators (2006). Effectiveness of clozapine versus olanzapine, quetiapine, and risperidone in patients with chronic schizophrenia who did not respond to prior atypical antipsychotic treatment. Am J Psychiatry.

[B18] Liu-Seifert H, Adams DH, Kinon BJ (2005). Discontinuation of treatment of schizophrenia patients is driven by poor symptom response: a pooled post-hoc analysis of four atypical antipsychotics drugs. BMC Med.

[B19] Weiden PJ, Olfson M (1995). Cost of relapse in schizophrenia. Schizophr Bull.

[B20] Gilmer TP, Dolder CR, Lacro JP, Folsom DP, Lindamer L, Garcia P, Jeste DV (2004). Adherence to treatment with antipsychotic medication and health care costs among Medicaid beneficiaries with schizophrenia. Am J Psychiatry.

[B21] Sun SX, Liu GG, Christensen DB, Fu AZ (2007). Review and analysis of hospitalization costs associated with antipsychotic nonadherence in the treatment of schizophrenia in the United States. Curr Med Res Opin.

[B22] Marcus SC, Olfson M (2008). Outpatient antipsychotic treatment and inpatient costs of schizophrenia. Schizophr Bull.

[B23] Tunis SL, Johnstone BM, Kinon BJ, Barber BL, Browne RA (2000). Designing naturalistic prospective studies of economic and effectiveness outcomes associated with novel antipsychotic therapies. Value Health.

[B24] Tunis SL, Faries DE, Nyhuis AW, Kinon BJ, Ascher-Svanum H, Aquila R (2006). Cost-effectiveness of olanzapine as first-line treatment for schizophrenia: results from a randomized, open-label, 1-year trial. Value Health.

[B25] Overall JE, Gorham DR (1962). The brief psychiatric rating scale. Psychol Rep.

[B26] Correll CU, Malhotra AK, Kaushik S, McMeniman M, Kane JM (2003). Early prediction of antipsychotic response in schizophrenia. Am J Psychiatry.

[B27] Leucht S, Busch R, Hamann J, Kissling W, Kane JM (2005). Early-onset hypothesis of antipsychotic drug action: a hypothesis tested, confirmed and extended. Biol Psychiatry.

[B28] Leucht S, Busch R, Kissling W, Kane JM (2007). Early prediction of antipsychotic nonresponse among patients with schizophrenia. J Clin Psychiatry.

[B29] Ramsey S, Willke R, Briggs A, Brown R, Buxton M, Chawla A, Cook J, Glick H, Liljas B, Petitti D, Reed S (2005). Good research practices for cost-effectiveness analysis alongside clinical trials: the ISPOR RCT-CEA Task Force report. Value Health.

[B30] Doshi JA, Glick HA, Polsky D (2006). Analyses of cost data in economic evaluations conducted alongside randomized controlled trials. Value Health.

[B31] Rosenheck RA, Leslie DL, Sindelar J, Miller EA, Lin H, Stroup TS, McEvoy J, Davis SM, Keefe RS, Swartz M, Perkins DO, Hsiao JK, Lieberman J, CATIE Study Investigators (2006). Cost-effectiveness of second-generation antipsychotics and perphenazine in a randomized trial of treatment for chronic schizophrenia. Am J Psychiatry.

[B32] Fu AZ, Dow WH, Liu GG (2007). Propensity Score and Difference-in-difference Methods: A Study of Second Generation Antidepressant Use in Patients with Bipolar Disorder. Health Services and Outcomes Research Methodology.

[B33] Senn S, Chow SC (2000). Crossover Design. Encyclopedia of Biopharmaceutical Statistics.

